# The impact of genetic counselling on risk perception and mental health in women with a family history of breast cancer

**DOI:** 10.1038/sj.bjc.6690139

**Published:** 1999-02

**Authors:** M Watson, S Lloyd, J Davidson, L Meyer, R Eeles, S Ebbs, V Murday

**Affiliations:** Royal Marsden NHS Trust and Institute of Cancer Research, UK; Mayday University Hospital, UK; St George's Hospital Medical School, UK

## Abstract

The present study investigated: (1) perception of genetic risk and, (2) the psychological effects of genetic counselling in women with a family history of breast cancer. Using a prospective design, with assessment pre- and post-genetic counselling at clinics and by postal follow-up at 1, 6 and 12 months, attenders at four South London genetic clinics were assessed. Participants included 282 women with a family history of breast cancer. Outcome was measured in terms of mental health, cancer-specific distress and risk perception. High levels of cancer-specific distress were found pre-genetic counselling, with 28% of participants reporting that they worried about breast cancer ‘frequently or constantly’ and 18% that worry about breast cancer was ‘a severe or definite problem’. Following genetic counselling, levels of cancer-specific distress were unchanged. General mental health remained unchanged over time (33% psychiatric cases detected pre-genetic counselling, 27% at 12 months after genetic counselling). Prior to their genetics consultation, participants showed poor knowledge of their lifetime risk of breast cancer since there was no association between their perceived lifetime risk (when they were asked to express this as a 1 in x odds ratio) and their actual risk, when the latter was calculated by the geneticist at the clinic using the CASH model. In contrast, women were more accurate about their risk of breast cancer pre-genetic counselling when this was assessed in broad categorical terms (i.e. very much lower/very much higher than the average woman) with a significant association between this rating and the subsequently calculated CASH risk figure (*P*= 0.001). Genetic counselling produced a modest shift in the accuracy of perceived lifetime risk, expressed as an odds ratio, which was maintained at 12 months' follow-up. A significant minority failed to benefit from genetic counselling; 77 women continued to over-estimate their risk and maintain high levels of cancer-related worry. Most clinic attenders were inaccurate in their estimates of the population risk of breast cancer with only 24% able to give the correct figure prior to genetic counselling and 36% over-estimating this risk. There was some improvement following genetic counselling with 62% able to give the correct figure, but this information was poorly retained and this figure had dropped to 34% by the 1-year follow-up. The study showed that women attending for genetic counselling are worried about breast cancer, with 34% indicating that they had initiated the referral to the genetic clinic themselves. This anxiety is not alleviated by genetic counselling, although women reported that it was less of a problem at follow-up. Women who continue to over-estimate their risk and worry about breast cancer are likely to go on seeking unnecessary screening if they are not reassured. © 1999 Cancer Research Campaign


*British Journal of Cancer* (1999) **79**(5/6), 868–874
© 1999 Cancer Research Campaign
Article no. bjoc.1998.0139

### The impact of genetic counselling on risk perception and mental health in women with a family history of breast cancer

**M Watson[1], S Lloyd[1], J Davidson[1], L Meyer[1], R Eeles[1], S Ebbs[2] and V Murday[3]**

[1]Royal Marsden NHS Trust and Institute of Cancer Research, [2]Mayday University Hospital, [3]St George's Hospital Medical School,

**Summary** The present study investigated: (1) perception of genetic risk and, (2) the psychological effects of genetic counselling in women with a family history of breast cancer. Using a prospective design, with assessment pre- and post-genetic counselling at clinics and by postal follow-up at 1, 6 and 12 months, attenders at four South London genetic clinics were assessed. Participants included 282 women with a family history of breast cancer. Outcome was measured in terms of mental health, cancer-specific distress and risk perception. High levels of cancer-specific distress were found pre-genetic counselling, with 28% of participants reporting that they worried about breast cancer 'frequently or constantly' and 18% that worry about breast cancer was 'a severe or definite problem'. Following genetic counselling, levels of cancer-specific distress were unchanged. General mental health remained unchanged over time (33% psychiatric cases detected pre-genetic counselling, 27% at 12 months after genetic counselling).

Prior to their genetics consultation, participants showed poor knowledge of their lifetime risk of breast cancer since there was no association between their perceived lifetime risk (when they were asked to express this as a 1 in x odds ratio) and their actual risk, when the latter was calculated by the geneticist at the clinic using the CASH model. In contrast, women were more accurate about their risk of breast cancer pre-genetic counselling when this was assessed in broad categorical terms (i.e. very much lower/very much higher than the average woman) with a significant association between this rating and the subsequently calculated CASH risk figure ($P = 0.001$). Genetic counselling produced a modest shift in the accuracy of perceived lifetime risk, expressed as an odds ratio, which was maintained at 12 months' follow-up. A significant minority failed to benefit from genetic counselling; 77 women continued to over-estimate their risk and maintain high levels of cancer-related worry.

Most clinic attenders were inaccurate in their estimates of the population risk of breast cancer with only 24% able to give the correct figure prior to genetic counselling and 36% over-estimating this risk. There was some improvement following genetic counselling with 62% able to give the correct figure, but this information was poorly retained and this figure had dropped to 34% by the 1-year follow-up. The study showed that women attending for genetic counselling are worried about breast cancer, with 34% indicating that they had initiated the referral to the genetic clinic themselves. This anxiety is not alleviated by genetic counselling, although women reported that it was less of a problem at follow-up. Women who continue to over-estimate their risk and worry about breast cancer are likely to go on seeking unnecessary screening if they are not reassured.

Provision of genetic counselling to women with a family history of breast cancer marks a fairly new development in oncology with the aim of educating individuals about their risk and encouraging those at increased risk to engage in health management strategies. The recent cloning of breast/ovarian cancer susceptibility genes (BRCA1 and BRCA2) is likely to increase demands on these clinical services (Miki et al, 1994; Wooster et al, 1995). There is controversy surrounding genetic counselling for women with a family history of breast cancer. The benefits of available risk management options are equivocal (with the exception of mammography in women aged 50 or over which is known to reduce deaths from breast cancer). It is not clear whether genetic counselling helps assuage cancer-related worries or has a beneficial effect on women's health.

In relation to mental health, women at risk of hereditary breast cancer may bear a heavy emotional burden (Lloyd et al, 1996) due to their familial experiences of life-threatening illness, high bereavement rates and fears of developing breast cancer. Growing evidence suggests a minority may have prolonged difficulties which undermine their mental health. A US evaluation of genetic counselling services indicates that 27% of clinic attenders have levels of distress consistent with the need for psychological support (Kash et al, 1992), and results from a population-based study of high-risk women show that over a third suffer from significant levels of worry about breast cancer (Lerman et al, 1991). Psychological responses such as these may undermine the effectiveness of genetic counselling and interfere with uptake of risk management recommendations.

In addition to the mental health issues it is not clear whether women understand the genetic information given or can make use of this in a way that would be beneficial to their mental or physical health. Current practice in genetic counselling is to convey risk information numerically, either as a risk of developing the disease per year, or risk by a certain age. There is some indication that aspects of genetic information may be poorly understood. Green (1978) suggested that the *qualitative* aspect of risk is more important than the *quantitative* and Leonard et al (1972) claim that

*Received 17 June 1998*
*Accepted 3 August 1998*

*Correspondence to:* M Watson, Psychological Medicine, Royal Marsden Hospital, Downs Road, Sutton, Surrey SM2 5PT, UK

people having genetic counselling 'are bad at probabilistic reasoning and find quantitative risk estimates difficult to understand'. If cancer family clinics are to provide a useful service it would be important to ensure that people understand, and can use, the risk information and the advice given. Lack of understanding may impact negatively on their ability to use this information when making decisions about the future management of their health and may affect their mental health if cancer-related worries are increased through some misunderstanding of the information.

The present study set out to examine these issues prospectively in a series of women with a family history of breast cancer attending four South London Cancer Family Clinics for genetic counselling. The study provides data on clinic attenders' risk perception, general psychological morbidity and cancer-specific worry.

### METHODS

### Participants

A consecutive series of 303 female first-time genetic clinic attenders was invited to participate. Accrual took place over 18 months, at four South London genetic counselling centres [Royal Marsden NHS Trust Hospital (two separate clinics), Mayday University Hospital and St Georges' Hospital], and local ethical committees approved the study. Inclusion criteria were: a family history of breast cancer, never clinically affected by cancer, no known serious mental illness, age 18 years and over, and able to complete a questionnaire.

### Procedure

Assessment was by self-administered questionnaires given at the genetic clinic immediately pre- and post-genetic consultation and by postal survey at 1, 6 and 12 months' follow-up.

### Outcome measures

Questionnaires were selected for validity, reliability and prior application to this population.

#### *Mental health*

***General Health Questionnaire*** Goldberg and Williams (1988) (pre-genetic counselling, 1, 6, and 12 months, follow-up). A brief, 12-item screening instrument assessing psychiatric disorder in non-psychiatric populations and previously used with medical patients.

***State-trait anxiety inventory*** Spielberger (1983) (pre- and post-genetic counselling). State version only, to monitor levels of anxiety at the clinic.

#### *Cancer-specific distress*

***Cancer Anxiety and Helplessness Scale*** Described by Kash (1992) (pre-genetic counselling, 1, 6 and 12 months' follow-up). This measures women's general cancer anxiety and feelings of helplessness in relation to cancer and cancer treatment.

***Impact of Event Scale*** Horowitz et al (1979) (pre-genetic counselling and 12 months' follow-up). Originally developed to determine levels of distress in response to a specific traumatic event. A modified version of this questionnaire, which has previously been used to gather information on cancer-specific distress in high-risk and general population women was included to assess psychological response, with specific reference to thoughts about risk of breast cancer over the last 7 days (Kash et al, 1992). Indices are provided on the extent to which the women experience intrusive and avoidant thoughts about breast cancer risk.

***Worry Scale*** Derived from Lerman and Schwartz (1993) (pre-genetic counselling and 12 months' follow-up). Two items were selected monitoring frequency of worry about cancer and the degree to which worry was perceived as a problem.

#### *Perception of risk (pre- and post-genetic counselling, and 12 months)*

Items assessed knowledge of: i) own *lifetime* chances of breast cancer based on the family history (expressed as a 1 in x odds ratio), ii) *relative* risk (chances of developing breast cancer compared with the average woman, on a 5-point scale, from 'very much lower' to 'very much higher than average'), iii) breast cancer incidence in the general population (1 in x). These items were previously developed and validated using other hereditary breast cancer populations (Lloyd et al, 1996).

#### *Clinic evaluation 'post-genetic counselling'*

Feedback was requested on the consultation and actions advised. Four-point rating scales assessed perception of clinic effectiveness, levels of reassurance derived from attendance and the extent to which information given was perceived to be helpful or worrying.

#### *Other measures*

Information was collected prospectively on health behaviours (these data will be reported fully in a further paper) in relation to whether women practiced breast self-examination, had read any leaflets on breast awareness or had had a mammogram. Responses on these items, assessed at the clinics' pre-genetic counselling (i.e. baseline data) are included in the present analysis of factors predicting cancer worry.

### Statistical methods

The $\chi^2$ statistic was used to test for evidence of association between categorical variables. Psychological scores at each time-point were summarized using mean, standard deviations (SD) or median, Inter-Quartile Range (IQR) as appropriate, and statistical tests based on parametric or non-parametric methods as necessary. Differences from baseline are presented in terms of mean (SD) and tests based on parametric methods. Differences for ordinal categorical data items were assessed using the Wilcoxon signed rank sum statistic. Where required, non-parametric Spearman Correlation Coefficients were calculated. Stepwise logistic regression was used to explore predictors of cancer worry. In computing the subscales for each psychological score on the questionnaires, all items were required to be present for an overall score to be calculated. The absence of one or more items from a particular subscale resulted in that score being deemed missing. All analyses were carried out using the SPSS release 4.0 package.

### RESULTS

Of the 303 participants complying with study entry criteria, ten eligible women were not approached due to clinic time constraints

© Cancer Research Campaign 1999

**Table 1** Demographics by hospital clinic

| | RMH London n = 42 (%) | RMH Sutton n = 45 (%) | Mayday n = 74 (%) | St George's n = 121 (%) | Total n = 282 (%) |
|---|---|---|---|---|---|
| *Marital status* | | | | | |
| Married/cohabiting | 29 (69) | 35 (78) | 58 (78) | 82 (68) | 204 (72) |
| Single | 8 (19) | 5 (11) | 8 (11) | 26 (21) | 47 (17) |
| Divorced/separated/widowed | 5 (12) | 5 (11) | 8 (11) | 13 (11) | 31 (11) |
| *Currently employed* | | | | | |
| Yes | 30 (71) | 29 (64) | 46 (62) | 81 (67) | 186 (66) |
| No | 12 (29) | 16 (35) | 28 (38) | 40 (33) | 96 (34) |
| *Social class* | | | | | |
| I | 6 (14) | 6 (13) | 7 (9) | 8 (7) | 27 (10) |
| II | 14 (33) | 13 (29) | 23 (31) | 36 (30) | 86 (30) |
| IIIN | 9 (21) | 3 (7) | 13 (18) | 23 (19) | 48 (17) |
| IIIM | 6 (14) | 14 (31) | 21 (28) | 29 (24) | 70 (25) |
| IV | 2 (5) | 1 (2) | 2 (3) | 5 (4) | 10 (4) |
| V | 1 (2) | 1 (2) | 2 (3) | 2 (2) | 6 (2) |
| Unclassified | 4 (10) | 7 (16) | 6 (8) | 18 (15) | 35 (12) |
| Median age (range) | 32 (22–55) | 34 (19–61) | 41 (28–59) | 39 (22–76) | 37 (19–76) |
| [a]Risk < 1 in 5 | 33 (79) | 32 (73) | 27 (39) | 39 (33) | 131 (48) |

[a]High risk relative to general population.

and a further ten declined the invitation to participate, comprising an overall cohort of 283. One participant was excluded due to missing baseline data, leaving a total of 282. Participants at each hospital did not differ significantly on demographic variables (i.e. social class, marital status and current employment) (Table 1). Most of the sample (186 participants, 66%) were currently employed, 204 (72%) were married/cohabiting and 161 (57%) were white collar/non-manual workers, as determined by the Office of Population Censuses and Surveys classification system (HMSO, 1980).

The age range of participants was 19–76 years, median 37 years. Women who attended the Royal Marsden NHS Trust Hospital (RMH) clinics were generally of younger age than those attending Mayday and St George's Hospitals (Kruskal–Wallis one-way analysis of variance (ANOVA), $P = 0.002$). Breast cancer risk was calculated by the clinical geneticists using the CASH model (Claus et al, 1991) based on the number of breast cancer cases in first- and second-degree relatives, the age of family members at disease onset and age of the woman presenting for genetic counselling. Women attending the RMH clinics had a higher risk of breast cancer as determined by the CASH model ($P < 0.001$; 1 in 5 or greater). Response rate (i.e. the percentage of the sample who completed and returned the questionnaires) was 96% (272/282) immediately post-counselling and, for postal follow-up, was 88% (249/282) at 1 and 6 months and rose to 93% (263/282) at 12 months.

Women were asked how they came to be referred to the clinic; 89/262 (34%) indicated that they had initiated the referral themselves. Of these, 55 had approached their General Practitioner (GP) asking specifically for information about genetic risk and 34 had approached their GP requesting direct access to screening on the basis of their family history. However, the majority of women (66%) were referred as the result of recommendation by a GP or hospital doctor or nurse. Other sources of referral included the well woman/family planning clinics, research channels and attending the clinic via a relative's appointment. No differences in mental health status were detected between the referral groups on any of the mental health measures used.

### Mental health

Using a cut-off of 3 points or more on the General Health Questionnaire (GHQ) to determine psychiatric caseness, a threshold previously applied to general practice samples on the basis that it achieves a balance between sensitivity and specificity (May, 1992), one-third of participants had notable levels of distress. A comparison of GHQ scores indicated no statistically significant change in general mental health at each follow-up compared to the pre-genetic counselling level (Table 2). Neither were there any statistically significant changes in levels of cancer-specific distress as measured by the Cancer Anxiety and Helplessness or the Impact of Event Scales.

Follow-up assessment revealed that 35/268 (13%) of the sample had received some psychological intervention during the 12 months since attending the genetic clinic. Of these, 19 (7%) had received psychotropic medication, ten (4%) had engaged in psychological counselling and six (2%) had received both forms of intervention.

Levels of state anxiety (Spielberger measure) pre-genetic counselling (mean 38.7, SD 10.5) were at a similar level to those reported in healthy women attending for breast screening (Morris and Greer, 1982). There was a significant downward shift in state anxiety immediately post-genetic counselling (mean 35.2, SD 10.8, $P < 0.001$).

Prior to genetic counselling, over a quarter (28%) of the sample stated that they worried about developing breast cancer 'frequently or constantly' and 18% felt that their breast cancer-related worry was a 'definite or severe problem' (Table 3). At 1-year follow-up, breast cancer worry remained at a similarly high level (23%), but there was a reduction (12%) in the extent to which this worry was perceived to be a problem ($P = 0.01$).

 © Cancer Research Campaign 1999

**Table 2** Measures of mental health – difference from baseline

| | Baseline | | | 1 month | | | | | 6 months | | | | | 12 months | | | | |
|---|---|---|---|---|---|---|---|---|---|---|---|---|---|---|---|---|---|---|
| | *n* | Mean | (SD) | *n* | Mean | (SD) | 95% CI | *P*-value[a] | *n* | Mean | (SD) | 95% CI | *P*-value[a] | *n* | Mean | (SD) | 95% CI | *P*-value[a] |
| General Health Questionnaire | 276 | 2.14 | (2.92) | 238 | −0.10 | (3.10) | −0.49,0.30 | 0.63 | 242 | −0.36 | (3.72) | −0.11,0.84 | 0.13 | 249 | −0.13 | (3.74) | −0.60,0.33 | 0.58 |
| Cancer Anxiety | 276 | 10.26 | (3.02) | 241 | 0.02 | (2.25) | −0.27,0.31 | 0.86 | 243 | 0.02 | (2.42) | −0.29,0.33 | 0.90 | 249 | −0.17 | (2.55) | −0.49,0.14 | 0.30 |
| Cancer Helplessness | 272 | 12.01 | (3.75) | 210 | 0.23 | (3.02) | −0.18,0.64 | 0.26 | 233 | 0.09 | (3.18) | −0.32,0.50 | 0.67 | 248 | 0.38 | (3.34) | −0.04,0.79 | 0.008 |
| Impact of Events Scale | | | | | | | | | | | | | | | | | | |
| Intrusion | 276 | 7.91 | (7.29) | | – | | – | – | | – | | – | – | 244 | −0.14 | (6.09) | −0.93,0.65 | 0.72 |
| Avoidance | 269 | 9.67 | (9.54) | | – | | – | – | | – | | – | – | 232 | −0.19 | (7.95) | −1.24,0.86 | 0.72 |
| Total | 267 | 17.52 | (15.70) | | – | | – | – | | – | | – | – | 229 | −0.29 | (12.45) | −1.91,1.33 | 0.73 |

[a]One sample *t*-test.

**Table 3** Breast cancer worry

| | Frequency (%) Baseline (*n* = 282) | 12 months (*n* = 257) | *P*-value[a] |
|---|---|---|---|
| How often do you worry about breast cancer? | | | |
| Not at all | 26 (9.2) | 27 (10.5) | |
| Occasionally | 177 (62.8) | 170 (66.3) | |
| Frequently | 61 (21.6) | 49 (19.0) | *P* = 0.09 |
| Constantly | 18 (6.4) | 11 (4.3) | |
| How much of a problem is breast cancer worry? | | | |
| Not at all | 81 (28.7) | 87 (33.7) | |
| Somewhat | 150 (53.2) | 140 (54.7) | |
| Definitely | 41 (14.5) | 23 (8.9) | *P* = 0.01 |
| Severe problem | 10 (3.5) | 7 (2.7) | |

[a]Wilcoxon signed rank.

### Risk perception

Table 4 indicates that the correct figure (expressed as an odds ratio) for lifetime risk was reported by only 25 (9%) women pre-genetic counselling but this rose to 84 (31%) reporting a correct odds ratio immediately post-genetic counselling. However, by 1 year the number reporting their risk correctly had dropped to 44 (17%). The correlation between women's perceived lifetime risk (i.e. reported as an odds ratio) assessed pre-genetic counselling, and the CASH risk figures (also an odds ratio) calculated by the geneticist at the clinic, was not significant ($r_s = 0.12$, $P = 0.09$), suggesting that women had poor prior knowledge of their numerical chances of breast cancer when asked to express this in terms of an odds ratio. Following genetic counselling this association strengthened (post-counselling, $r_s = 0.60$, $P < 0.001$) and was maintained at 12 months, but at a lower level ($r_x = 0.30$ $P < 0.001$). Ratings of lifetime risk (CASH) and relative risk were associated modestly pre-counselling and strengthened at follow-up (pre: $r_s$=-0.19, p = 0.002; post: $r_s$ = -0.50 $P < 0.001$; 12 months; $r_s$ = 0.40, $P < 0.001$). Figure 1 shows the proportions of women who over-, under-, or correctly estimated their lifetime risk at each time point. In this analysis perceived over- and under-estimation of risk was defined as any response greater or less than the stated CASH risk figure, respectively. This appears to show some overall improvement post counselling, with a fall off in improvement at 1 year. However, the pattern of individual changes from baseline is interesting. One hundred and three (57%) of the 182 women who were able to give an estimate of personal risk at each time point remained unchanged in their risk perception, 38 (21%) were previously incorrect but at 12 months gave the right figure. However, 18 (10%) who were correct pre-counselling later provided an inaccurate risk estimate at 12 months. The remaining 11% moved between over- and under-estimating or 'don't know'. Of the 126 who over-estimated their lifetime risk pre-genetic counselling and who also responded at 1 year, 77 (61%) continued to overestimate. Although those who over-estimated their risk were no different in terms of general mental health (GHQ) from those who estimated correctly, or underestimated, their risk they reported significantly higher cancer-specific distress pre-genetic counselling and at 12 months' follow-up ($P < 0.001$ for both avoidance and intrusion subscales of the Impact of Event Scale at each time point). A greater proportion of them worried 'frequently' or 'constantly' about breast cancer at the 12-month follow-up; 43% persistent

*© Cancer Research Campaign 1999*

**Table 4** Estimation of personal risk

| | Post-counselling | | | | | |
|---|---|---|---|---|---|---|
| | **Over-estimate** | **Correct** | **Under-estimate** | **Don't Know** | **Missing** | **Total** |
| Baseline Response | | | | | | |
| Over-estimate | 67 | 40 | 20 | 4 | 7 | 138 |
| Correct | 4 | 13 | 8 | 0 | 0 | 25 |
| Under-estimate | 12 | 17 | 16 | 0 | 3 | 48 |
| Don't know | 12 | 14 | 21 | 6 | 4 | 57 |
| Missing | 1 | 0 | 0 | 1 | 12 | 14 |
| Total | 96 | 84 | 65 | 26 | 11 | 282 |
| | **12 months** | | | | | |
| | **Over-estimate** | **Correct** | **Under-estimate** | **Don't Know** | **Missing** | **Total** |
| Baseline Response | | | | | | |
| Over-estimate | 77 | 21 | 22 | 6 | 12 | 138 |
| Correct | 8 | 6 | 10 | 0 | 1 | 25 |
| Under-estimate | 9 | 9 | 20 | 5 | 5 | 48 |
| Don't know | 14 | 8 | 16 | 10 | 9 | 57 |
| Missing | 0 | 0 | 0 | 2 | 12 | 14 |
| Total | 108 | 44 | 68 | 39 | 23 | 282 |

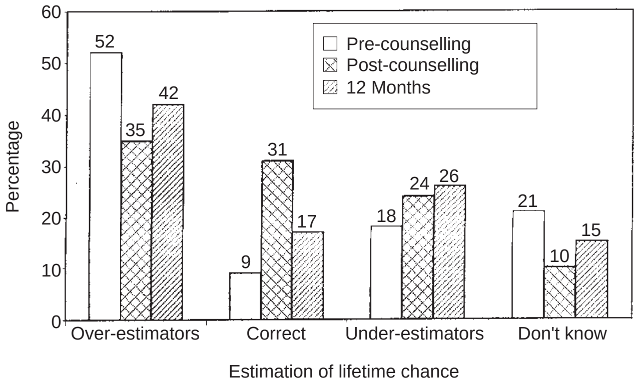

**Figure 1** Risk perception (odds ratio)

over-estimators versus 22% others ($P = 0.002$). These results indicate that a substantial minority of women with specific worries about cancer remain fixed in their own over-estimation of risk and their worry about breast cancer. Those individuals who under-estimated their risk prior to genetic counselling did not show increased breast cancer worry once informed of their CASH risk. There were no differences on the Cancer Anxiety and Helplessness Scale according to whether correct or incorrect cancer risk estimates were given by the women. When women were asked, pre-genetic counselling, to rate their risk of breast cancer relative to the average woman, the majority 151/279 (54%) felt their chances were 'somewhat higher than average', and this perception remained unchanged with time (post-counselling 144/266 (54%); 12 months 132/255 (52%)). Perceived risk relative to the average woman was significantly negatively correlated with the geneticists' calculated risk figure both before and after clinic attendance, i.e. a high perceived relative risk was correlated with a high CASH risk figure (pre-genetic counselling; r = -0.21, $P$ < 0.001; post-genetic counselling: r = -0.43, $P$ < K 0.01; 12 months:, r = -0.31, $P$ < 0.001). Pre-counselling, 55% (143/260) perceived their relative risk correctly; this increased slightly to 63% (154/250) post-counselling and 61% (145/249) at 1 year, but not significantly so ($P$ < 0.10 in each case). At 12 months women perceiving their risk to be very much higher than average were significantly more likely to report intrusive thoughts about risk of breast cancer ($P = 0.01$).

Evaluation of women's estimates of breast cancer incidence in the general population indicated they were largely inaccurate pre-genetic counselling; only 68/282 (24%) gave the correct 1 in 12 figure and 101 (36%) over-estimated. In contrast, 167/269 (62%) gave the correct 1 in 12 figure immediately post-counselling but this dropped to 87/258 (34%) correct at 12 months. Women's own perceived risk, given as an odds ratio, was significantly correlated with their general population estimate at each time point ($P$ < 0.001 in each case) suggesting that even if these figures were inaccurate they tended to relate to each other. However, a comparison between estimates of general population and own risk (odds ratio) showed that women who got the general population figure correct pre-counselling were not more likely to get their own risk correct than those who gave an inaccurate population risk figure.

In summary, specific figures about risk, provided within the genetic consultation, tend not to be remembered by these women. Following genetic counselling a significant minority of women either continue to incorrectly estimate risk or shift their risk estimate in an inaccurate direction. Continual over-estimators may be worrying unnecessarily and excessively about breast cancer risk and under-estimators appear undisturbed by the information that their risk is greater than they thought. Specific defence mechanisms in the latter women may limit their intake of threatening information. Under-estimators were not significantly different from the rest of the sample in terms of their scores for intrusive and avoidant thoughts about breast cancer risk (as measured on the Impact of Events Scale) when this was assessed pre-counselling. However, at 12 months' follow-up their scores were significantly lower than the rest of the sample on each of these scales (avoidance $P = 0.02$; intrusion $P = 0.006$) indicating that in the long-term they are less likely to report having intrusive thoughts about breast cancer risk.

*© Cancer Research Campaign 1999*

### Predictors of cancer worry

Age, perceived risk, actual risk (CASH figure), practice of breast self-examination, having had a mammogram, and having read leaflets on breast awareness were used in the modelling procedures as potential predictors of cancer worry at baseline (categorized as not at all/occasionally = 0, frequently/constantly = 1). Baseline Impact of Event Scale and state anxiety scores were not included as they are so highly correlated with cancer worry. Both at baseline and at 1 year the only variable seen to have a significant predictive effect was perceived risk – i.e. it is how women perceive their risk that predicts cancer worry rather than actual risk. Those who either under-estimated or correctly perceived their risk were less likely to worry frequently or constantly about cancer than those who over-estimated (under-estimators' odds ratio (OR) = 0.3, 95% confidence interval (CI) = 0.1, 1.0; correct estimators' OR = 0.5, 95% CI = 0.2, 1.0, $P = 0.02$ (trend) pre-counselling; under-estimators' OR = 0.4, 95% CI =0.2, 1.0; correct estimators' OR = 0.2, 95% CI = 0.1, 0.5, $P < 0.01$ (trend) at 1 year).

Comparable results are obtained when using women's ratings of risk relative to the average woman as their perceived risk. Further models using how much cancer worry is perceived to be a problem as the outcome variable also gave similar results.

### Clinic evaluation

Attitude toward the clinical service was generally favourable. The majority 252/272 (92%) reported that the clinical had been 'moderately' or 'extremely' effective and when asked how reassuring they felt the consultation had been, 80% indicated that it was 'moderately' or 'extremely' reassuring, and only a small minority (5%), that it was 'not at all reassuring'. Twenty per cent of women felt the consultation was moderately or extremely worrying but the majority (93%) perceived the clinic as 'moderately or extremely' helpful.

### DISCUSSION

Evidence from this prospective study of genetic clinic attenders indicates that there are high levels of cancer-related worry that compare unfavourably to previously gathered data on general population risk samples (Lloyd et al, 1996). The finding that genetic counselling fails to alleviate this cancer-specific distress in a substantial minority of women is contrary to previous US findings (Kash et al, 1992), reporting a reduction in cancer anxiety several months post-genetic counselling. However, a single genetic counselling consultation may not be sufficient to shift these worries in some women and it might be unreasonable to expect otherwise. General levels of psychological morbidity (GHQ) remain unaffected by genetic counselling and are consistent with those previously reported elsewhere in the literature (Watson et al, 1998).

In relation to risk perception and worry about cancer, the data show that women who consistently over-estimate their breast cancer risk are most vulnerable to cancer-specific worry. These women represent a group that can be targeted by clinicians for psychological support. Such women may constitute a drain on breast screening services by requesting unnecessary mammograms and clinical examinations. Psychological support intended to help alleviate their worries may be more appropriate than breast surveillance given that the majority of these women are too young to enter the UK national mammographic screening programme. A notable finding, in line with results reported by Evans et al (1994), is that there is a group of women who underestimate their risk and when given a higher risk estimate by the clinical geneticist do not show any immediate increase in cancer-related worry. The most likely explanation is that their perception of risk is unaltered by the genetic consultation, therefore worry is not triggered because they continue to underestimate their risk. These constitute an important group for further investigation. It would be important to know the impact of risk under-estimation on subsequent level of uptake of methods for managing their increased risk of breast cancer.

It seems reasonable to assume that women would tend not to be aware of specific odds ratio risk figures prior to genetic counselling, given that they would be unlikely to consider their risk in these statistical terms. However, genetic counselling should bring some change in the tendency for women to under- or over-estimate their risk since an aim of the consultation is to correct their estimates of risk where necessary. The service produced some immediate improvement in women's knowledge of their risk figure (a correct odds ratio being quoted by 31% post-counselling compared to 9% pre-counselling), but by 1 year follow-up the number of women giving the correct figure had dropped back to 17%. This suggests that specific numerical genetic risk information is less salient to these women than their own risk beliefs, which were not shifted significantly by the genetic consultation; 57% providing a risk estimate pre-counselling remained unchanged in their risk perception. However, the majority were generally in the right 'ballpark' for risk with their more general perceptions relative to the average woman; 61% were correct in this risk estimate at the 12-month follow-up even though they were poor at giving specific numerical details. This is in contrast with our previous finding (Lloyd et al, 1996) where no association was found between CASH calculation and women's perception of their risk relative to the general population. However, we previously used a 3-point rating scale of relative risk which may have been too crude to distinguish these differences in risk perception.

Women attending the clinic were largely inaccurate in their reporting of the incidence of breast cancer in the general population with only a small proportion (24%) able to give the correct 1 in 12 statistic. In relation to informing women, through genetic counselling, about the general population risk of breast cancer, participants were better able to give the correct figure immediately post-counselling but this information was not retained and had returned to approximately the same level as pre-counselling 1 year later.

The impact of increasing numbers of women developing breast cancer in the general population over the last 3 decades and the attention the media pays to it may play some role in some of this worry. Many women will now have the experience of having family members with breast cancer and will wish to know whether there is a genetic predisposition. Only a minority may have a familial predisposition to breast cancer and need to be referred to specialist genetic services.

### Clinical implications

Genetic counselling produced some limited improvement in women's understanding of their specific numerical risk of breast cancer. Many had a general view of their risk relative to the average woman which was accurate. Of more concern is the substantial minority who did not benefit from genetic counselling

© Cancer Research Campaign 1999

because they continued to over-estimate their risk and their worry about developing breast cancer was unrelieved. A further small group of women who under-estimated their risk may have failed to benefit in terms of future management of their health because they continued to under-estimate risk following the consultation.

Resources may need to be available to provide psychological support where genetic risk counselling fails to alleviate high levels of cancer-specific distress and future investigation should be directed towards examining the value of integrating this into the service offered. This may require some broadening in the training of genetic counsellors and associates to provide them with additional psychological skills, along with integrating mental health professionals into the genetic teams in a liaison capacity. The majority of women participating in the investigation attended prior to the availability of genetic tests for the BRCA1 and BRCA2 cancer predisposition genes. Since genetic testing is already underway and may raise cancer-related distress in gene carriers (Watson et al, 1996) the investigation of efficacious methods of appropriate psychological support is becoming more pressing.

This study highlights some problems in the provision of cancer genetic counselling. Some women continue to believe they are at high risk despite being told otherwise and point to a number of ‘worried well’ getting drawn into the system. Many of these women could probably be managed by general practitioners at the primary care level rather than within specialist genetic services. Overall, there is a need to develop better ways of imparting information so that women understand their risk and how to manage it. A clear programme of how to deal with genetic risk from the primary care level through to tertiary services needs to be developed.

### ACKNOWLEDGEMENTS

We are grateful to all the women who willingly gave their time to complete questionnaires, and to the Cancer Research Campaign (CRC project CP1026) for their support of this study. We would also like to thank the following for their contribution: Ms D Averill, Dr A Brady, Mrs S Gray, Dr K Kash, Prof B Ponder and Ms J Quillam.

### REFERENCES

Claus EB, Risch NJ and Thompson WD (1991) Genetic analysis of breast cancer in the cancer and steroid hormone study. *Am J Hum Genet* **48**: 232–242

Evans DGR, Blair V, Greenhalgh R, Hopwood P and Howell A (1994) The impact of genetic counselling on risk perception in women with a family history of breast cancer. *Br J Cancer* **70**: 934–938

Goldberg D and Williams P (1988) *A User’s Guide to the General Health Questionnaire*. NFER-Nelson: Windsor, UK

Green CH and Brown RA (1978) Counting lives. *J Occ Accidents* **2**: 55

Horowitz M, Wilner N and Alvarez W (1979) Impact of events scale: a measure of subjective stress. *Psychosom Med* **41**: 209–218

Kash KM, Holland JC, Halper MS and Miller DG (1992) Psychological distress and surveillance behaviours of women with a family history of breast cancer. *J Natl Cancer Inst* **84**: 24–30

Leonard C, Chase G and Child B (1972) Genetic counselling, a consumer’s view. *N Engl J Med* **287**: 433

Lerman C and Schwartz M (1993) Adherence and psychological adjustment among women at high risk for breast cancer. *Breast Cancer Res Treat* **28**: 145–155

Lerman C, Trock B, Rimer B, Boyce A, Jepson C and Engstrom P (1991) Psychological and behavioral implications of abnormal mammograms. *Ann Int Med* **114**: 657–661

Lloyd S, Watson M, Waites B, Meyer L, Eeles R, Ebbs S and Tylee A (1996) Familial breast cancer: a controlled study of risk perception, psychological morbidity and health beliefs in women attending for genetic counselling. *Br J Cancer* **74**: 482–487

May S (1992) Patient satisfaction and the detection of psychiatric morbidity in general practice. *Family Practice* **9**: 76–81

Morris T and Greer S (1982) Psychological characteristics of women electing to attend a breast screening clinic. *Clin Oncol* **8**: 113–119

Miki Y, Swensen J, Shattuck-Eidens D, Futreal A, Harshman K, Tavtigian S and Lui Q (1994) A strong candidate for the breast and ovarian cancer susceptibility gene BRCA1. *Science* **266**: 66–71

Spielberger CD (1983) *State-Trait Anxiety Inventory for Adults*. Consulting Psychologists’ Press: Palo Alto, CA

Watson M, Lloyd S, Eeles R, Ponder B, Easton D, Seal S, Averill D, Daly P, Ormiston W and Murday V (1996) Psychosocial impact of testing (by linkage) for the BRCA1 breast cancer gene: an investigation of two families in the research setting. *Psycho-oncology* **5**: 233–239

Watson M, Duvivier V, Wade Walsh M, Ashley S, Papaikonomou M, Eeles R, Sacks N and Murday V (1998) Family history of breast cancer: what do women understand and recall about their genetic risk? *J Med Genetics* **35**: 731–738

Wooster R, Bignell G, Lancaster J, Swift S, Seal S, Mangion J, et al (1995) Identification of the breast cancer susceptibility gene BRCA2. *Nature* **378**: 789–792

 © Cancer Research Campaign 1999
